# Representation of American Indian and Alaska Native Individuals in Academic Medical Training

**DOI:** 10.1001/jamanetworkopen.2021.43398

**Published:** 2022-01-13

**Authors:** Lala L. Forrest, Brooks P. Leitner, Cirila Estela Vasquez Guzman, Erik Brodt, Charles A. Odonkor

**Affiliations:** 1 Frank H. Netter MD School of Medicine, Quinnipiac University, North Haven, Connecticut; 2 Medical Scientist Training Program, Yale School of Medicine, New Haven, Connecticut; 3 Family Medicine, Oregon Health Science University, Portland; 4 Family Medicine and Northwest Native American Center of Excellence, Oregon Health Science University, Portland; 5 Division of Physiatry, Department of Orthopedics and Rehabilitation, Yale School of Medicine, Orthopedics and Rehabilitation, Interventional Pain Medicine and Physiatry, Yale New Haven Hospital, New Haven, Connecticut

## Abstract

**Importance:**

Identifying gaps in inclusivity of Indigenous individuals is key to diversifying academic medical programs, increasing American Indian and Alaska Native representation, and improving disparate morbidity and mortality outcomes in American Indian and Alaska Native populations.

**Objective:**

To examine representation of American Indian and Alaska Native individuals at different stages in the 2018-2019 academic medical training continuum and trends (2011-2020) of American Indian and Alaska Native representation in residency specialties.

**Design, Setting, and Participants:**

A cross-sectional, population-based analysis was conducted using self-reported race and ethnicity data on trainees from the Association of American Medical Colleges (2018), the Accreditation Council for Graduate Medical Education (2011-2018), and the US Census (2018). Data were analyzed between February 18, 2020, and March 4, 2021.

**Exposures:**

Enrolled trainees at specific stages of medical training.

**Main Outcomes and Measures:**

The primary outcome was the odds of representation of American Indian and Alaska Native individuals at successive academic medical stages in 2018-2019 compared with White individuals. Secondary outcomes comprised specialty-specific proportions of American Indian and Alaska Native residents from 2011 to 2020 and medical specialty–specific proportions of American Indian and Alaska Native physicians in 2018. Fisher exact tests were performed to calculate the odds of American Indian and Alaska Native representation at successive stages of medical training. Simple linear regressions were performed to assess trends across residency specialties.

**Results:**

The study data contained a total of 238 974 607 White and American Indian and Alaska Native US citizens, 24 795 US medical school applicants, 11 242 US medical school acceptees, 10 822 US medical school matriculants, 10 917 US medical school graduates, 59 635 residents, 518 874 active physicians, and 113 168 US medical school faculty. American Indian and Alaska Native individuals had a 63% lower odds of applying to medical school (odds ratio [OR], 0.37; 95% CI, 0.31-0.45) and 48% lower odds of holding a full-time faculty position (OR, 0.52; 95% CI, 0.44-0.62) compared with their White counterparts, yet had 54% higher odds of working in a residency specialty deemed as a priority by the Indian Health Service (OR, 1.54; 95% CI, 1.09-2.16). Of the 33 physician specialties analyzed, family medicine (0.55%) and pain medicine (0.46%) had more than an average proportion (0.41%) of American Indian and Alaska Native physicians compared with their representation across all specialties.

**Conclusions and Relevance:**

This cross-sectional study noted 2 distinct stages in medical training with significantly lower representation of American Indian and Alaska Native compared with White individuals. An actionable framework to guide academic medical institutions on their Indigenous diversification and inclusivity efforts is proposed.

## Key Points

**Question:**

How does representation of American Indian and Alaska Native individuals at specific stages of academic medical training compare with representation of their White counterparts?

**Findings:**

This cross-sectional study including 1.35 million American Indian and Alaska Native and White individuals in each stage of the 2018-2019 academic medical training continuum found that, compared with their White peers, American Indian and Alaska Native individuals had 63% lower odds of applying to medical school compared with the general US population and 48% lower odds of holding a full-time faculty position postresidency.

**Meaning:**

These findings suggest there are distinct stages in academic medical training for targeted policy and program changes to increase the representation of American Indian and Alaska Native individuals.

## Teaser

This cross-sectional study examines the representation of American Indian and Alaska Native individuals through the academic stages of medical training and proposes a framework that may be used to enhance diversification efforts.

## Introduction

The resurgence of the Black Lives Matter movement and the disparate health impact of the COVID-19 pandemic on communities of racial and ethnic minority groups have galvanized medical schools and residency programs to re-examine their role in advancing health equity in which individuals have a fair and just opportunity to be as healthy as possible.^1^ Given that 25% of American Indian and Alaska Native individuals die before the age of 45 years compared with 15% for African American and 7% for White individuals,^2^ inclusion of Indigenous-specific efforts is necessary for MD-granting institutions to fully advance health equity.

Increasing American Indian and Alaska Native representation in the biomedical workforce has been a strategy to address American Indian and Alaska Native health inequities.^2^ Studies show that racial and ethnic concordance between patients and physicians is associated with higher patient satisfaction, better communication, greater use of preventive services, and increased report of receiving all needed medical care.^3,4,5,6,7,8^ In addition, physicians of racial and ethnic minority groups are more likely to work in underserved areas and treat more racial and ethnic minority patients.^5^ Because racial and ethnic identity are associated with quality of care and patient satisfaction, there exists a need for the health care workforce to better reflect the national and local populations in which individuals receive care.

Although current efforts are geared toward increasing the number of underrepresented individuals of racial and ethnic minority groups in medicine, these strategies have fallen short of benefitting American Indian and Alaska Native individuals.^9,10,11,12,13,14,15^ Reasons for this lack of progress are myriad and include the paucity of American Indian and Alaska Native mentors, lack of cultural and tribal community integration,^16,17,18^ and poor understanding of the unique barriers that exist for American Indian and Alaska Native students,^19^ among others.^2^ Efforts have been made to address some of the underlying problems, for example, by creating American Indian and Alaska Native–specific pathway programs.^13,20^ Recognition of the pervasiveness of racism across the entire academic and research enterprise has even led to calls to move beyond traditional ways of thinking based on prima facie assumptions of meritocracy in the academy.^21,22^ Simultaneously, there is movement toward integration of antiracism in graduate medical education to combat institutionalized barriers underlying current inequities in medical training.^23^ Part of this move has spurred a focused analysis of the various stages of training in an attempt to understand where barriers persist and to find potential solutions.^24^ The literature is scant on gaps in inclusivity of Indigenous individuals at specific stages of training, despite ample evidence of global American Indian and Alaska Native underrepresentation.^2,25,26^ Although various transitional support programs have been designed to increase representation of American Indian and Alaska Native trainees at different stages of medical training: precollege, college, medical school preapplication, postbaccalaureate, residency, and faculty development^9,10,11,13,15,20,27,28,29^ (eTable in the Supplement), few original research reports have evaluated the efficacy and outcomes of American Indian and Alaska Native representation in medicine.^25,26,30^

To address this knowledge deficit, we sought to examine each stage of academic medical training to ascertain where disparities exist for American Indian and Alaska Native trainees compared with their White peers. The overarching theme was to identify areas of opportunity in the continuum for programmatic development such that all future medical trainees would be well equipped to meet the health needs of American Indian and Alaska Native communities. Because of the limited research in this area, our primary objective was to evaluate the odds of American Indian and Alaska Native representation at successive stages of training to identify gaps that can be strategically targeted for American Indian and Alaska Native–specific academic development. Making advances in inclusive health equity for Indigenous individuals begins with an evidence-based approach that moves beyond the statistics of low numbers of American Indian and Alaska Native individuals in medicine that have been documented for decades.^31^ Instead, we aim to provide an overview of the current status of American Indian and Alaska Native representation up to the faculty level, examine trends of American Indian and Alaska Native trainee specialty choice, and the current active American Indian and Alaska Native physician workforce.

## Methods

Data analysis was performed between February 18, 2020, and March 4, 2021. The Yale University Institutional Review Board approved the study (February 11, 2020). Waiver of informed consent was granted as per Yale University Protocol for Exemption Request category 4 (secondary research uses of identifiable private information, where the identifiable private information or identifiable biospecimens are publicly available). This study followed the Strengthening the Reporting of Observational Studies in Epidemiology (STROBE) reporting guideline for observational studies.

### Data Sources

All data sources used in this study are publicly available. The most recently available data sets were used in each instance and data for each stage of training were pulled from the most appropriate available source, listed as described herein. To analyze US medical school applicants, matriculants, and graduates, American Indian and Alaska Native physicians by specialty, and White and American Indian and Alaska Native medical school faculty, we used 2018-2019 data from the Association of American Medical Colleges (AAMC).^32^ Resident data by race, ethnicity, and specialty were obtained from the Accreditation Council for Graduate Medical Education (ACGME),^33^ as well as the 2020 data from the AAMC.^34^ Data were obtained for all applicable academic years, from 2011 to 2020. Demographic characteristics were obtained from the US Census 2018 database.^35^ Racial and ethnicity data in all sources are self-reported.

The Indian Health Service (IHS) is an agency within the Department of Health and Human Services and serves as the main federal health care provider for American Indian and Alaska Native individuals across 574 federally recognized tribes. Residency specialties deemed as a medical priority by the IHS (ie, residencies that will have the greatest influence on advancing American Indian and Alaska Native health)^36^ were evaluated in this study and included internal medicine, pediatrics, emergency medicine, surgery, obstetrics and gynecology, anesthesiology, family medicine, and psychiatry (combined residency programs were excluded from our analysis to prevent double counting that could potentially alter relative proportions between specialties). The longitudinal trend analysis divided these IHS priority residencies into 3 broad subsets (surgical-, medical-, or hospital-based residencies) as characterized by 2013 ACGME data.^37^

### Outcomes

The primary outcome was the odds of representation of American Indian and Alaska Native individuals at successive stages of training compared with the representation of White individuals. Secondary outcome measures were the odds of an American Indian and Alaska Native individual working in an IHS priority residency compared with their White peers, the proportion of American Indian and Alaska Native individuals in IHS priority residency specialties, and the proportion of American Indian and Alaska Native physicians in 33 different medical specialties.

### Statistical Analysis

Two-sided Fisher exact tests were performed to compute an odds ratio (OR) and 95% CI. Odds ratios reflect the relative proportions of American Indian and Alaska Native and White trainees in each stage of the 2018-2019 academic medical training continuum; a lower OR means that American Indian and Alaska Native individuals are disproportionately underrepresented at the second stage compared with their relative representation at the first (in each comparison) stage and does not reflect individuals throughout their training. Proportions are shown as the American Indian and Alaska Native or White proportion of the total category population. A simple linear regression was computed and the slope of a line of best fit was compared with 0 for significance to examine trends across residency specialties. All data analyses were conducted in GraphPad Prism, version 8.0 (GraphPad Software). Choropleth maps were created in the Advanced US Data Map Excel Add-In. A 2-sided *P* value <.05 was determined to be statistically significant.

## Results

### Participant Data

The study data contained a total of 238 974 607 White and American Indian and Alaska Native US Citizens, 24 795 US medical school applicants, 11 242 US medical school acceptees, 10 822 US medical school matriculants, 10 917 US medical school graduates, 59 635 residents, 518 874 active physicians, and 113 168 US medical school faculty (Table).

**Table.** Demographic Characteristics of American Indian and Alaska Native and White Individuals in Each Stage of the 2018-2019 Academic Medical Training Continuum^a^

Characteristic	Individuals, No. (% of total)		Total No. of individuals
	White	American Indian and Alaska Native	
US census population	236 173 020 (73.1)	2 801 587 (0.9)	322 903 031
US medical school			
Applicants	24 686 (46.8)	109 (0.2)	52 777
Acceptees	11 198 (49.8)	44 (0.2)	22 483
Medical school			
Matriculants	10 783 (49.9)	39 (0.2)	21 622
Graduates	10 879 (54.6)	38 (0.2)	19 937
Residents	59 359 (42.3)	276 (0.2)	140 391
Active physicians	516 304 (56.2)	2570 (0.3)	918 547
Full-time faculty members	112 894 (63.9)	274 (0.2)	176 732

^a^ Data were obtained from the American Association of Medical Colleges,^34^ US Census Bureau,^35^ and Accreditation Council for Graduate Medical Education.^37^

### Odds of Representation During Medical Training

**Figure 1.**
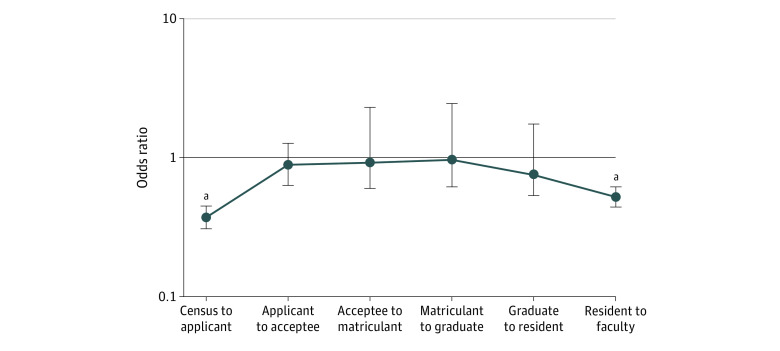
Odds of American Indian and Alaska Native Alone Trainee Representation at Successive Medical Stages Compared With White Counterparts in 2018-2019 Odds were calculated by the Fisher exact test from the 2 continuum categories listed at each measurement point for American Indian and Alaska Native individuals compared with White individuals. Odds ratios are displayed on a log scale. Error bars indicate 95% CI. Data were obtained from the American Association of Medical Colleges,^34^ US Census Bureau,^35^ and Accreditation Council for Graduate Medical Education.^37^ ^a^*P* < .001.

Figure 1 shows the odds of an American Indian and Alaska Native individual (designated as American Indian and Alaska Native alone) being represented in the next stage of medical training compared with their White peers. American Indian and Alaska Native individuals from the general US population had 63% lower odds of applying to medical school compared with their White counterparts (OR, 0.37; 95% CI, 0.31-0.45; *P* < .001). Once American Indian and Alaska Native individuals applied to medical school, their odds of being offered acceptance (OR, 0.89; 95% CI, 0.63-1.26; *P* = .54), matriculating (OR, 0.92; 95% CI, 0.60-2.30; *P* = .74), graduating (OR, 0.97; 95% CI, 0.62-2.46; *P* = .91), and working in a residency program (OR, 0.75; 95% CI, 0.53-1.75; *P* = .10) did not differ significantly from those of their White peers. However, American Indian and Alaska Native residents had 48% lower odds of faculty representation in comparison with White residents (OR, 0.52; 95% CI, 0.44-0.62; *P* < .001) and had 54% higher odds of working in a residency deemed as a health priority for Indigenous communities by the IHS. States with the highest proportion of American Indian and Alaska Native medical school applicants were not associated with the states with the highest proportion of American Indian and Alaska Native individuals (eFigure 1 in the Supplement).

### Proportion in IHS Priority Residency Specialties

**Figure 2.**
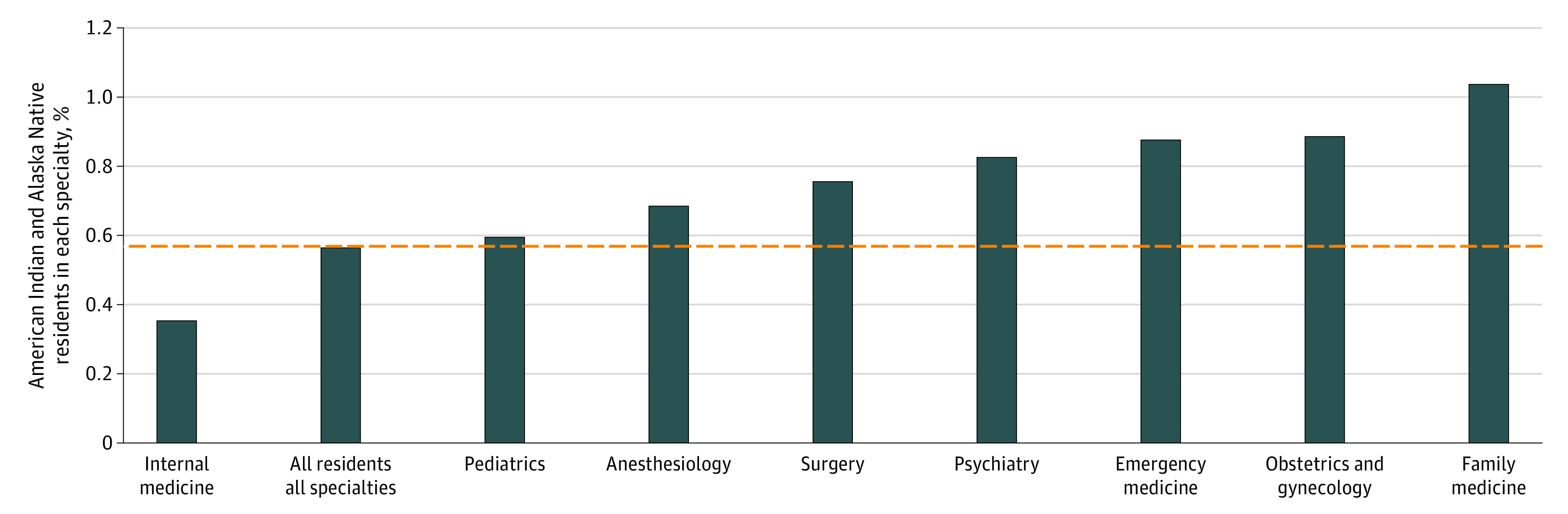
Proportion of All American Indian and Alaska Native Alone or in Combination Residents in the Indian Health Service Priority Residencies in 2019-2020 The 2018-2019 Indian Health Service Commitment Handbook designates the 8 residencies as in need for American Indian and Alaska Native communities. Dual residency programs were omitted. The horizontal dashed line represents the proportion of American Indian and Alaska Native physicians in all specialties combined. Data were obtained from the American Association of Medical Colleges.^34^

Figure 2 shows the proportion of total American Indian and Alaska Native (designated as alone or in combination with another race) residents in the 8 residency programs deemed as a high priority by the IHS in 2019-2020. American Indian and Alaska Native individuals make up 0.57% of all residents in IHS priority residencies; family medicine had the highest proportion of American Indian and Alaska Native residents (1.04%), whereas internal medicine had the lowest (0.36%) proportion. In addition, American Indian and Alaska Native medical graduates had significantly higher odds of being in an IHS priority residency compared with their White peers (OR 1.54; 95% CI, 1.09-2.16; *P* = .02). eFigure 2 in the Supplement shows the discrepancies between the AAMC and ACGME databases on how American Indian and Alaska Native racial identity is reported.

### Trends in Residency Preference

**Figure 3.**
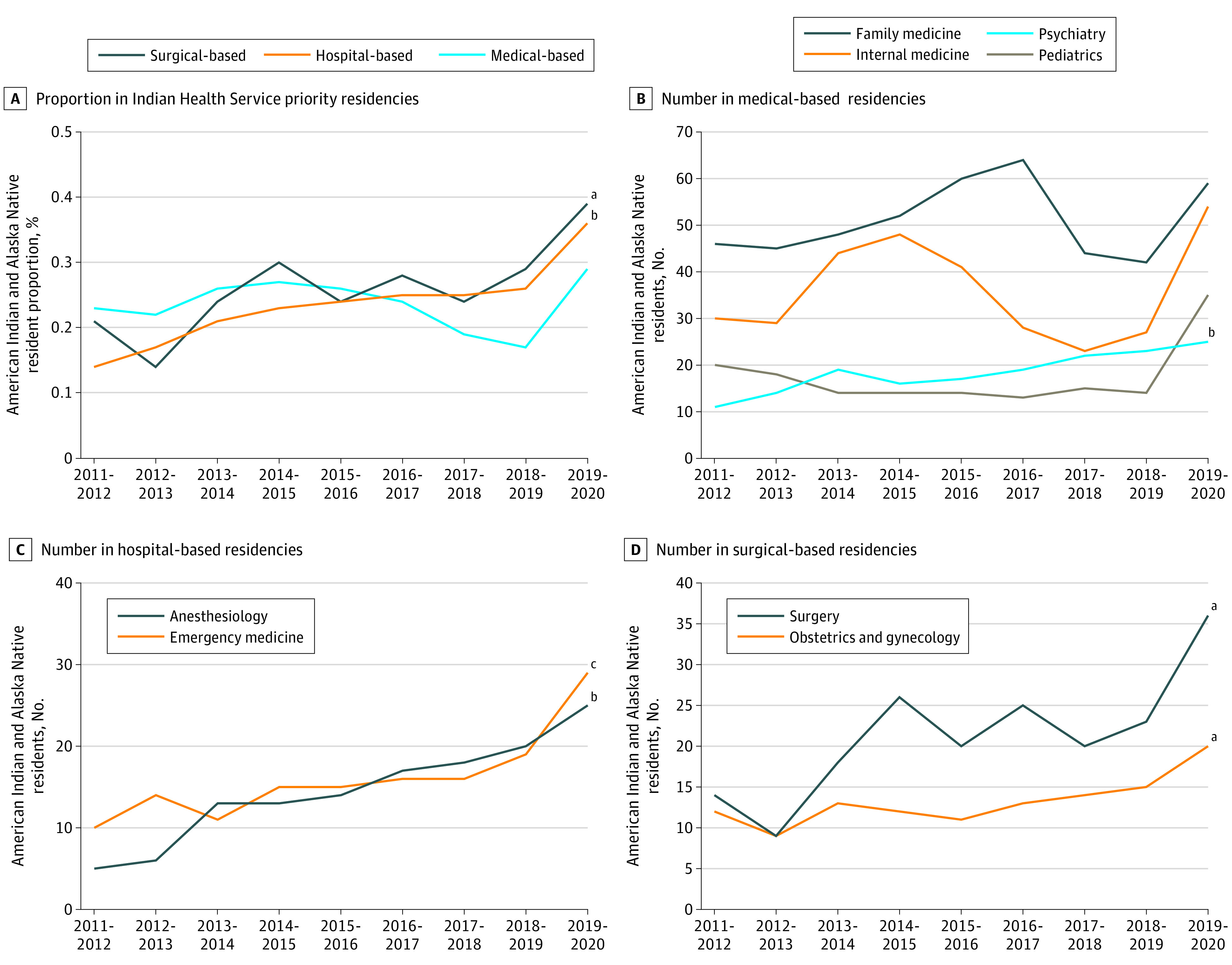
Trends in the Proportions of American Indian and Alaska Native Alone Resident Representation From 2011 to 2020 in Indian Health Service Priority Residencies The 2018-2019 Indian Health Service Commitment Handbook designates the 8 specialties as in need for American Indian and Alaska Native communities. Surgical (surgery and obstetrics and gynecology), medical (family medicine, internal medicine, pediatrics, and psychiatry), and hospital based (anesthesiology and emergency medicine) are categorized according to the Accreditation Council for Graduate Medical Education.^37^ ^a^*P* < .05. ^b^*P* < .001. ^c^*P* < .01.

Figure 3 shows the hospital-based residencies (anesthesiology and emergency medicine) and surgical-based residencies (general surgery and obstetrics and gynecology) have significantly increased their representation of American Indian and Alaska Natives from 0.14% of total hospital-based residents in 2011-2012 to 0.36% in 2019-2020 (slope, 0.02; *F* = 39.80; *P* < .001) and from 0.21% of total surgical-based residents in 2011-2012 to 0.39% in 2019-2020 (slope, 0.02; *F* = 9.74; *P* = .02). A 5-fold increase in the number of American Indian and Alaska Native resident trainees was observed in anesthesiology in the past 9 years (slope, 2.27; *F* = 120.20; *P* < .001) and an approximate 3-fold increase in the number of emergency medicine residents (slope, 1.70; *F* = 16.98; *P* < .01). Within the surgical-based category, there was a 2.5-fold increase in the number of general surgical American Indian and Alaska Native resident trainees (slope, 2.22; *F* = 11.55; *P* = .01) and nearly a 2-fold increase in obstetrics and gynecology (slope, 0.88; *F* = 11.40; *P* = .01). Psychiatry was the only medical-based residency to have significantly increased American Indian and Alaska Native representation across time (slope, 1.53; *F* = 51.55; *P* < .01).

### Specialty Choice of Active Physicians

**Figure 4.**
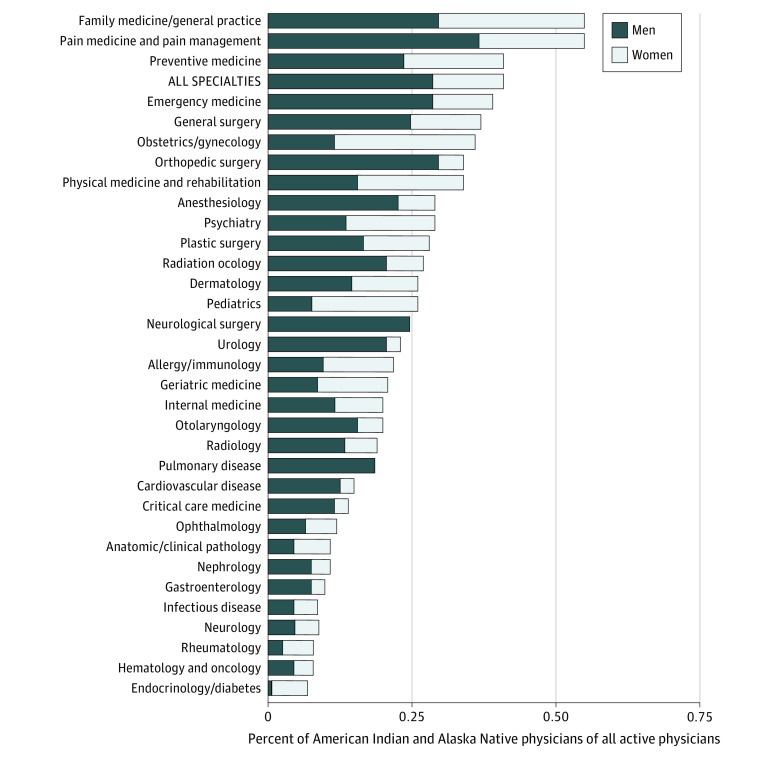
Proportion of Active Physicians in the US Who Identify as American Indian and Alaska Native Alone in 2018 by Specialty Specialties are in descending order from highest to lowest relative representation among all physicians of all races and ethnicities. Data were obtained from the American Association of Medical Colleges.^34^

Figure 4 shows the specialties of current practicing American Indian and Alaska Native physicians in 2018. Across all specialties, American Indian and Alaska Native individuals represented 0.41% of all physicians. Of the 33 physician specialties analyzed, only family medicine (0.55%) and pain medicine (0.46%) had higher American Indian and Alaska Native physician representation compared with their total representation across all specialties. American Indian and Alaska Native women were underrepresented in most specialties, with 29% of all American Indian and Alaska Native physicians being women. The specialties with the lowest representation of American Indian and Alaska Native physicians were in the medical subspecialties, including endocrinology (0.07%), hematology/oncology (0.08%), rheumatology (0.08%), infectious diseases (0.09%), gastroenterology (0.10%), nephrology (0.11%), internal medicine (0.20%), and cardiology (0.15%).

## Discussion

Using data from the AAMC, ACGME, and US Census, we highlight gaps in diversity with discordantly lower representation of American Indian and Alaska Native individuals at 2 key stages: application to medical school and retention/promotion to faculty. The findings add to the literature with an in-depth analysis that goes beyond general underrepresentation of American Indian and Alaska Native individuals compared with White individuals in medicine by highlighting specific gaps in the academic medical training continuum that underlie inequities in training and recruitment for American Indian and Alaska Native individuals.^38,39,40,41,42,43,44^ The data show a 63% lower odds of American Indian and Alaska Native individuals applying to medical school compared with their White counterparts in the 2018-2019 academic year. In addition, there was a 48% lower odds of an American Indian and Alaska Native trainee compared with their White counterparts holding a full-time faculty position after residency. The significant underrepresentation of American Indian and Alaska Native faculty may be multifactorial, including lack of effective faculty recruitment, promotion, and retention practices. Factors such as minority tax, imposter syndrome, biases in hiring and promotion, and others, are also involved.^45,46^ Furthermore, the lack of diverse faculty mentorship is a leading obstacle for medical trainees of minority racial and ethnic groups interested in pursuing a career in academic medicine.^47,48^ Our finding that White trainees are disproportionately overrepresented compared with American Indian and Alaska Native trainees indicates a need for re-examination of leadership advancement opportunities and faculty promotion policies. There is also a need to explore recruitment and develop best practices that will better support American Indian and Alaska Native individuals to enter and remain in academic medicine.^49^

In 2018, American Indian and Alaska Native medical school graduates had 54% higher odds of enrollment into an IHS priority residency. With American Indian and Alaska Native alone or in combination trainees composing 0.57% of the total residents in 2019-2020, we found that 7 of the 8 IHS priority residencies had higher than average American Indian and Alaska Native representation (>0.57%) compared with all specialties, with family medicine being the highest (1.04%). The one residency with less representation than average was internal medicine (0.36%). It remains to be determined whether this finding is attributed to ineffective recruitment practices from certain residency programs and/or American Indian and Alaska Native trainee specialty preference. There was an upward shift by American Indian and Alaska Native persons toward more surgical- and hospital-based residencies from 2011 to 2020, which sheds light on current and potential future gaps in care and is especially important for tribal communities in rural areas.^9,16,17^ We found a substantial increase in the representation of American Indian and Alaska Native residents in psychiatry and anesthesiology from 2011 to 2020. A study by Chen et al^50^ noted an increase in American Indian and Alaska Native psychiatry applicants in 2019 compared with 2014. Similar changes have been reported in another study.^51^ The American Psychiatric Association has made visible changes to promote diversity, equity, and inclusion practices, such as establishing the Division of Diversity and Healthy Equity and selecting Altha Stewart, MD, to become the first Black member to oversee the organization after 174 years.^52^ There is limited research in the field of anesthesiology on diversity, equity, and inclusion efforts as it pertains to recruitment and retention of American Indian and Alaska Native individuals. Thus, it remains to be determined how this specialty has attracted more American Indian and Alaska Native residents over the years.

The data reported herein indicate a mismatch between American Indian and Alaska Native patient needs vs existing specialist services. American Indian and Alaska Native physicians are predominantly practicing in the fields of family medicine, pain medicine, and preventive medicine. The top 5 leading causes of death for American Indian and Alaska Native individuals are heart disease, cancer, accidents, diabetes, and liver disease.^53^ It is important to emphasize that American Indian and Alaska Native active physicians are least represented among the fields of hematology and oncology (0.08%) and endocrinology (0.07%) and rank low in gastroenterology (0.10%), cardiology (0.15%), and internal medicine (0.20%). The dearth of American Indian and Alaska Native hematologists, oncologists, endocrinologists, gastroenterologists, and cardiologists potentially poses profound limitations in access to specialist services and may be further affecting the rates of use and/or access by American Indian and Alaska Native populations.^54^ The paucity of American Indian and Alaska Native specialists implies that American Indian and Alaska Native patients may be seeking specialty-level care from American Indian and Alaska Native family medicine practitioners, which suggests that these physicians may carry an overwhelming burden of patients with increasingly complex health conditions. In addition, there is debate on whether improvements in specialists, traditional medicine practitioners, and/or preventive care would yield the greatest health benefits in American Indian and Alaska Native populations.^55,56,57^ Beyond this, representation matters in helping minimize health inequities.^58^

We recommend the following framework in conjunction with a consensus statement^59^ for how academic medical institutions can amplify diversification efforts to advance American Indian and Alaska Native–inclusive health equity.

First, recruit and promote American Indian and Alaska Native faculty. This action prioritizes program sustainability and long-term American Indian and Alaska Native faculty development and advancement. This top-down approach illustrates the commitment of academic medical institutions to enhance the American Indian and Alaska Native medical trainee and faculty experience. Nearly 30% of White medical school faculty members have the full professor rank, compared with less than 5% of American Indian and Alaska Native faculty.^25^ It is necessary for institutions to move beyond outdated passive recruitment efforts, such as posting job descriptions and opportunities, to proactively reaching out to potential American Indian and Alaska Native candidates. The importance of a diverse faculty search committee cannot be overstated in efforts to broaden applicant pools. It is important to consider that diversifying search committees without first achieving greater numbers of diverse faculty may precipitate or exacerbate minority tax for fellow faculty members.^60^ After successfully recruiting American Indian and Alaska Native faculty, institutions can provide a supportive environment to foster American Indian and Alaska Native faculty success and ascension in academic rank. Institutions should recruit and consider American Indian and Alaska Native faculty with the appropriate credentials for chief diversity officer and faculty senate leadership positions.^61^ Active faculty development and training via intramural grants and research support for American Indian and Alaska Native faculty would likely facilitate career advancement and, in turn, mentorship of American Indian and Alaska Native trainees. Diversification of the faculty by increasing American Indian and Alaska Native faculty must be viewed as necessary to achieve academic excellence.

Second, invest in American Indian and Alaska Native–specific transition support programs in the academic medical training continuum. This study suggests that American Indian and Alaska Native–specific medical training development initiatives leading up to and at the medical school application stage may have the greatest return on investment. To meet the specific health needs of American Indian and Alaska Native communities, tribal community knowledge and guidance can help strengthen programming and unlock access to American Indian and Alaska Native youth. American Indian and Alaska Native–specific programs that strategically integrate local tribal communities and cultural mentors into the program infrastructure, as well as grant American Indian and Alaska Native participants the opportunity to study American Indian and Alaska Native disease disparity, have been shown to increase the number of American Indian and Alaska Native college graduates who successfully matriculate into medical school.^27^ These cultural and community elements help American Indian and Alaska Native students navigate cognitive dissonance as they learn to balance cultural identity inside and outside of the classroom. We recommend that US MD-granting institutions hold themselves accountable to their mission statements as it pertains to their constituencies, which includes American Indian and Alaska Natives, and invest in American Indian and Alaska Native–specific initiatives to increase the number of American Indian and Alaska Native physicians.

Third, it is necessary to learn from American Indian and Alaska Native trainees and physicians. Our data demonstrate a dearth of American Indian and Alaska Native physicians in internal medicine subspecialties with a shortage of specialists who treat the most serious conditions seen in American Indian and Alaska Native individuals. Furthermore, observed trends in medical specialty choice over the last decade indicate an exacerbation of the shortage of these specialists. To realign these trends to fit the needs of American Indian and Alaska Native communities, medical institutions can educate American Indian and Alaska Native and non-American Indian and Alaska Native individuals about these trends and study the factors that contribute to American Indian and Alaska Native trainee specialty preference. Dedicating resources to explore trainee specialty preference may help identify structural barriers that may be resolved to advance health equity in the Indigenous population.

### Limitations

This study has limitations. An important confounder to studying American Indian and Alaska Native representation in academia is the inconsistency across, and even within, academic organizations at defining and reporting American Indian and Alaska Native identity. According to the 2010 Census, nearly half of American Indian and Alaska Native persons reported being American Indian and Alaska Native in combination with at least 1 other race. A recent study found that the number of American Indian and Alaska Native applicants, matriculants, and graduates increased significantly when the American Medical College Application System allowed applicants to select more than 1 racial identity in 2002.^26^ This modification demonstrates the importance for databases to capture both American Indian and Alaska Native alone and American Indian and Alaska Native in combination to accurately measure American Indian and Alaska Native representation. The only public data that comprehensively count all American Indian and Alaska Native individuals (ie, individuals who identify as American Indian and Alaska Native alone and individuals who identify as American Indian and Alaska Native plus another race), are 2019-2020 residency data from the AAMC (eFigure 2 in the Supplement). We recommend that medical organizations continue to work on enhancing their racial and ethnicity measurements and to be inclusive of, and report on, Indigenous peoples who identify as more than 1 race. There needs to be consistent longitudinal racial and ethnic reporting at all levels of medical training to better understand changes occurring throughout the academic medical training continuum. Furthermore, a widespread challenge is that many data sets do not include American Indian and Alaska Native demographic information by sex or tribal affiliation, which are important considerations for upholding the principles of Indigenous data sovereignty.^23^

## Conclusions

This study provides a framework for how academic institutions can enhance American Indian and Alaska Native health equity by targeting program development at specific key stages of the academic medical training continuum. We found gaps in diversity with discordantly lower odds of progression for American Indian and Alaska Native individuals at 2 key stages: application to medical school and retention/promotion to faculty. The discordance between American Indian and Alaska Native subspecialty selection and the health needs of American Indian and Alaska Native communities warrants further evaluation of factors associated with subspecialty preference. Our data identify specific residency specialties that have been successful at diversity of Indigenous populations with proportionally higher rates of enrolling American Indian and Alaska Native trainees. Gaps in American Indian and Alaska Native diversity and inclusion present opportunities for MD-granting institutions to engage tribal community members and Indigenous organizations. These collaborations may help increase the number of American Indian and Alaska Native medical school applicants and faculty alike.

## Notes

Supplement. **eTable.** Pathway Programs Designed to Promote American Indian and Alaska Native Representation in Academic Medicine **eFigure 1.** Proportion of Each State’s Population or State’s Applicant Pool That Is American Indian and Alaska Native Alone **eFigure 2.** Proportion of American Indian and Alaska Native Residents in Indian Health Service Priority Residencies in 2019-2020, ACGME Versus AAMC
