# Supplementary material for: Representation of American Indian and Alaska Native Individuals in Academic Medical Training
Source: JAMA Netw Open. 2022 Jan 13;5(1):e2143398. doi: 10.1001/jamanetworkopen.2021.43398 (PMC8759009; doi:10.1001/jamanetworkopen.2021.43398)
Supplement: Supplement. — eTable. Pathway Programs Designed to Promote American Indian and Alaska Native Representation in Academic Medicine eFigure 1. Proportion of Each State’s Population or State’s Applicant Pool That Is American Indian and Alaska Native Alone eFigure 2. Proportion of American Indian and Alaska Native Residents in Indian Health Service Priority Residencies in 2019-2020, ACGME Versus AAMC [file jamanetwopen-e2143398-s001.pdf]

Supplementary Online Content

Forrest LL, Leitner BP, Vasquez Guzman CE, Brodt E, Odonkor CA. Representation of American Indian and Alaska Native individuals in academic medical training. *JAMA Netw Open*. 2022;5(1):e2143398. doi:10.1001/jamanetworkopen.2021.43398

eTable. Pathway Programs Designed to Promote American Indian and Alaska Native Representation in Academic Medicine

eFigure 1. Proportion of Each State's Population or State's Applicant Pool That Is American Indian and Alaska Native Alone

eFigure 2. Proportion of American Indian and Alaska Native Residents in Indian Health Service Priority Residencies in 2019-2020, ACGME versus AAMC

This supplementary material has been provided by the authors to give readers additional information about their work.

© 2022 Forrest LL et al. *JAMA Network Open.*

eTable. Pathway Programs Designed to Promote American Indian and Alaska Native Representation in Academic Medicine.

Stage of Academic Medical Training	Program	Year Began	Mission	Website
Pre-College	University of Wisconsin-Madison School of Medicine and Public Health's Indigenous Health and Wellness Day	2005	Promote and educate AIAN middle and high school students about higher education and health professional career opportunities	https://www.med.wisc.edu/education/native-american-center-for-health-professions/student-recruitment/
	Northwest Native American Center of Excellence Tribal Health Scholars	2017	Support and inspire AIAN high school students to enter health careers	https://www.nnacoe.org/tribal-health-scholars
	We R Native	2012	To provide comprehensive health resources for Native youth, by Native youth. We R Native strives to promote holistic health and positive growth in our local communities and nation at large	https://www.wernative.org/my-life/life-hacks/text-healers
	WE ARE HEALERS	2012	Inspire AIAN youth to see themselves as future health leaders through the stories of Native health professionals	https://wearehealers.org/
College	Indian Health Service's Indians Into Medicine Program (INMED) – Reimagine Indians into Medicine (RISE) Summery Academy at the WSU Elson S. Floyd College of Medicine	2020	6-week program that prepares 15 federally recognized AIAN individuals for the MCAT and medical school application process	https://medicine.wsu.edu/about-the-college/diversity-inclusion/pathway-programs/inmed-reimagine-indians-into-medicine-rise-pathway-program/
	University of Minnesota's Native Americans into Medicine Program	1973	6-week summer enrichment program for college sophomores and juniors interested in pursuing health careers	https://med.umn.edu/caimh/college-premed
	University of Utah's Native American Summer Research	2010	10-week summer program with the goal to support the academic, career, and personal development of Native American	https://medicine.utah.edu/pediatrics/research/education/nari/

© 2022 Forrest LL et al. *JAMA Network Open.*

Stage of Academic Medical Training	Program	Year Began	Mission	Website
	Internship (NARI)		students who are interested in Health Science careers.	
**Medical School Application**	Pacific West Collective (OHSU/WSU/UC-Davis/U-W) Medical School Applicant workshop	2018	A one day event designed for AIAN pre-medical students who are actively preparing to apply to medical school within the next two years	https://www.nnacoe.org/applicant-workshop
	Great Lakes Alliance (MN/WI/MSU/UND) Medical School Applicant workshop	2013	A multi-day workshop aimed at networking Native American pre-medical students from across the region to meet faculty and staff, as well as offer in-depth application tips and career development opportunities	N/A
	Four Corners Alliance Pre-Admissions Workshop	2011	To bring together AAIP member physicians, AIAN college students, public health professionals, university admissions professionals and other interested individuals with the goal of providing students with the information and skills necessary to succeed in the medical and health-profession school admission process	https://www.aaip.org/programs/student-programs/pre-admission-workshop/
**Post-Baccalaureate**	Oregon Health & Science University Wy'east Pathway	2018	Increase the number of AIANs who enter US MD-Granting institutions	https://www.nnacoe.org/wyeast-pathway
	University of Arizona Pre-Medical Admissions Pathway (PMAP)	2014	To facilitate the development of knowledge, skills, and attributes that are needed for successful medical students to become providers of high-quality healthcare for Arizona	https://medicine.arizona.edu/admissions/p-map
**Residency**	Seattle Indian Health Board	1994	To pass down decades of knowledge about, not only the practice of medicine, but also working with a community with unique needs—American Indians and Alaska Natives.	https://www.sihb.org/services-and-programs/residency-program/
	University of New Mexico Shiprock Rural Residency Program	2020	To increase the number of family physicians serving rural Native American communities. Ultimately the goal is to improve the health of Native Americans living in rural areas.	https://fcm.unm.edu/education/residency/shiprock-rural-residency-program.html

© 2022 Forrest LL et al. *JAMA Network Open.*

Stage of Academic Medical Training	Program	Year Began	Mission	Website
**Faculty**	Spirit of EAGLES	2000	To follow a community-based participatory approach, and work in partnership with tribes and urban Indian communities, multiple cancer centers, the American Cancer Society and many others to address American Indian/Alaska Native (AIAN) cancer prevention and control needs, combine research and action to improve health outcomes, and mentor new AIAN researchers and health professions students.	http://www.nativeamericanprograms.net/spirit-of-eagles/
	Oregon Health & Science University Indigenous Faculty Forum	2017	Promote the advancement and retention of Indigenous faculty in Academic Medicine	https://www.nnacoe.org/iff

© 2022 Forrest LL et al. *JAMA Network Open.*

eFigure 1. Proportion of Each State's Population or State's Applicant Pool That Is American Indian and Alaska Native Alone.

A

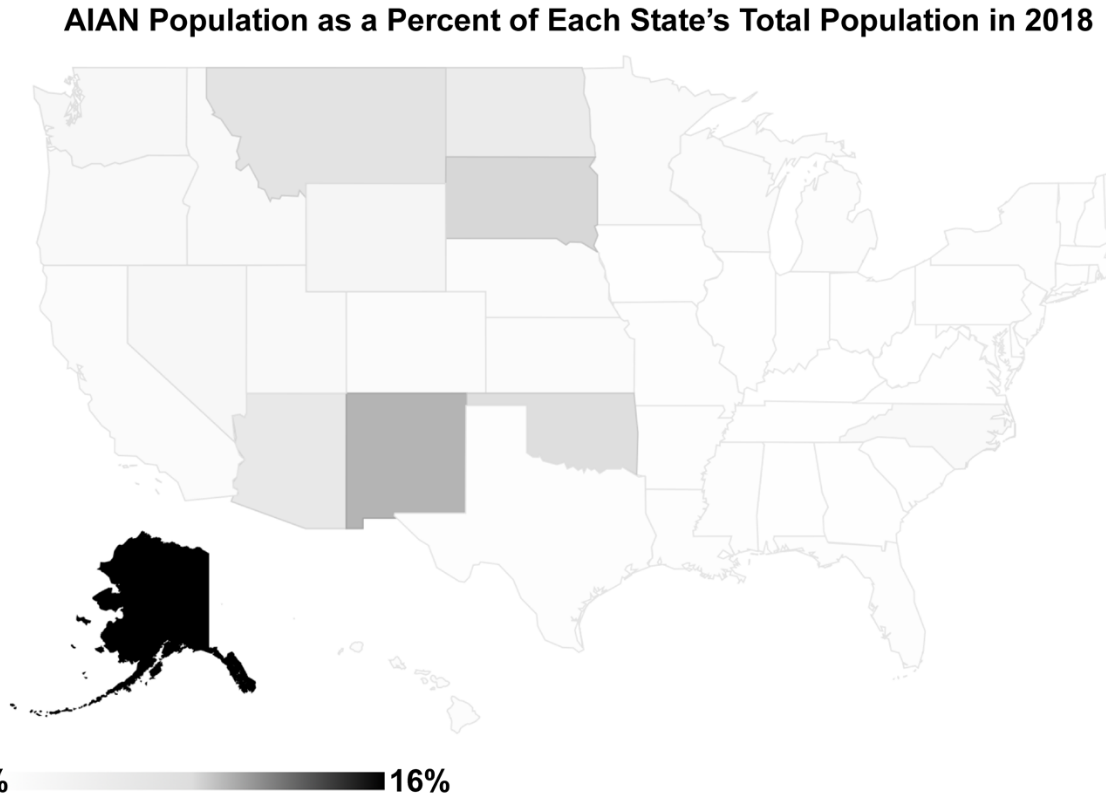

B

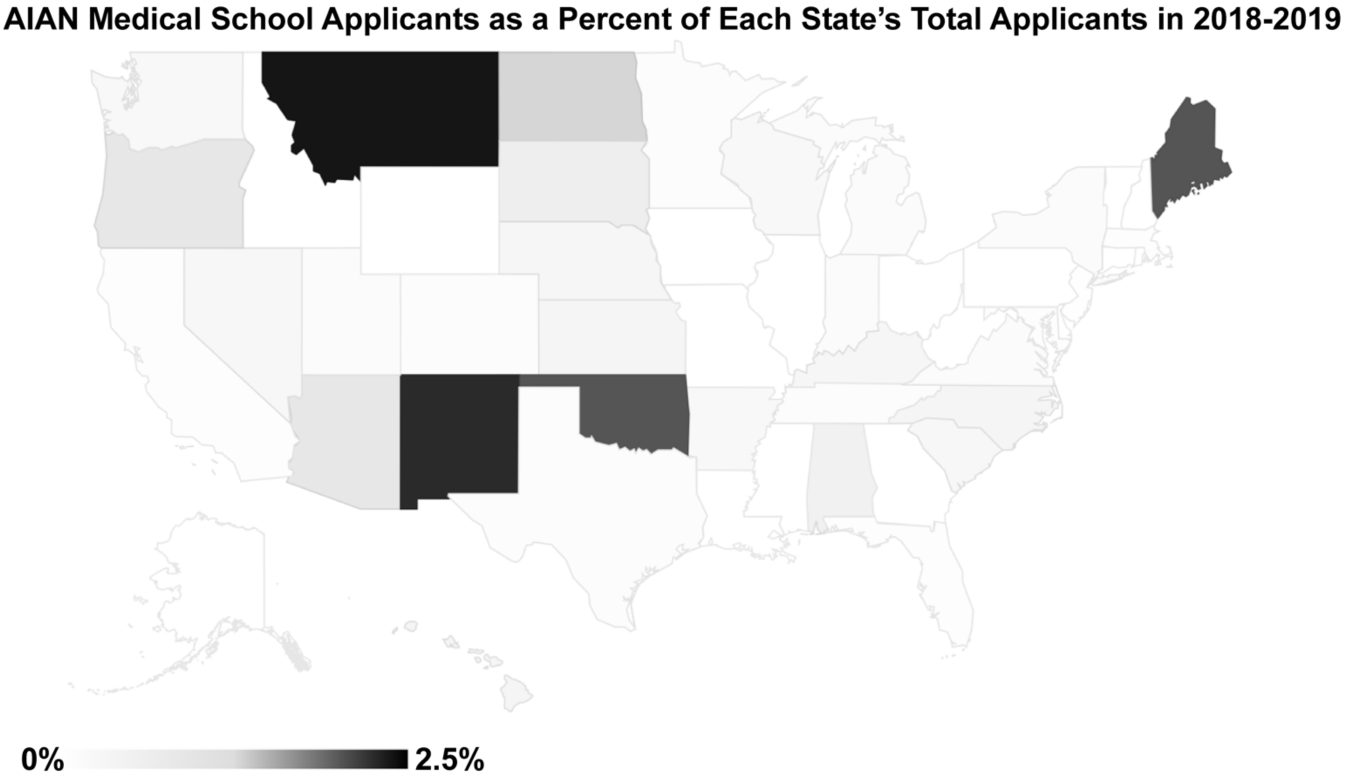

eFigure 1 in the Supplement: Proportion of Each State's Population or State's Applicant Pool That is American Indian and Alaska Native (AIAN) Alone. Choropleth maps were created in the Advanced US Data Map Excel Add-In.
Source: US Census Microdata Database 2018 and AAMC Diversity in Medicine: Facts and Figures 2019.

© 2022 Forrest LL et al. *JAMA Network Open.*

eFigure 2. Proportion of American Indian and Alaska Native Residents in Indian Health Service Priority Residencies in 2019-2020, ACGME versus AAMC.

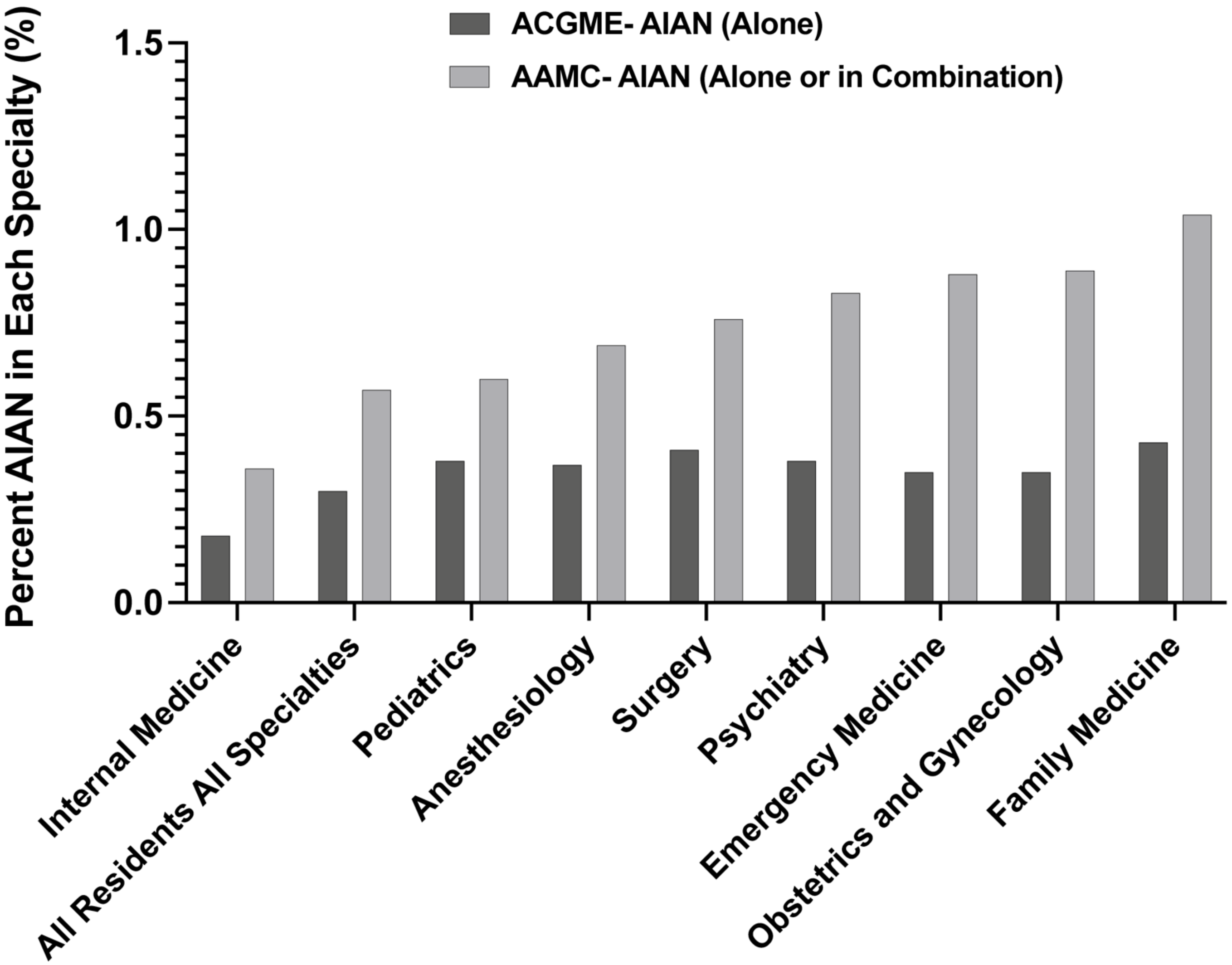

eFigure 2 in the Supplement: Proportion of American Indian and Alaska Native (AIAN) Alone and/or in Combination in the Indian Health Service Priority Residencies 2019-2020. Data collected from the ACGME is reported as AIAN-Alone while data form the AAMC is AIAN-Alone or in Combination. Source: Data obtained from ACGME Data Resource Book 2019-2020 and AAMC 2020 Report on Residents.

© 2022 Forrest LL et al. *JAMA Network Open.*
